# Minimally Invasive Extended Thymothymectomy With the “Lateral View Preceding Method”

**DOI:** 10.1016/j.atssr.2022.07.003

**Published:** 2022-08-07

**Authors:** Shohei Mori, Takamasa Shibazaki, Daiki Kato, Takeo Nakada, Mitsuo Yabe, Takashi Ohtsuka

**Affiliations:** 1 Division of Thoracic Surgery, Department of Surgery, The Jikei University School of Medicine, Tokyo, Japan

## Abstract

Robot-assisted thymothymectomy with 1 subxiphoid and 3 intercostal ports provides high maneuverability around the upper poles of the thymus and excellent visualization of the superior sections of the phrenic nerves. However, it limits the visibility of the inferior sections of the phrenic nerves and hinders maneuverability around the pericardiophrenic angle in cases of extended thymothymectomy. This report describes the “lateral view preceding method” that successfully uses bilateral thoracoscopy to dissect the lower poles of the thymus and mediastinal fat around the pericardiophrenic angle, followed by robot-assisted thymothymectomy to remove the upper poles of the thymus.

The Video can be viewed in the online version of this article [https://doi.org/10.1016/j.atssr.2022.07.003] on http://www.annalsthoracicsurgery.org

Video- and robot-assisted thoracic surgery is a minimally invasive procedure routinely used for mediastinal tumor resection and thymectomy.^1^^,^^2^ Irrespective of the technique used, various approaches, such as the patient’s body position (supine or lateral), with or without CO_2_ insufflation or sternal lifting, through the bilateral or unilateral thoracic cavity, and port site selection, are considered, each with its advantages and disadvantages.^2^, ^3^, ^4^, ^5^

Our institution (The Jikei University School of Medicine, Tokyo, Japan) uses robot-assisted thoracic surgery with 1 subxiphoid and 3 intercostal ports, a supine position for the patient, and CO_2_ insufflation for anterior mediastinal tumor resection and thymectomy. This combination provides high maneuverability around the upper poles of the thymus and left brachiocephalic vein (BCV) and excellent visualization of the superior sections of both the phrenic nerves. However, this procedure offers poor visibility of the inferior sections of the phrenic nerves and low maneuverability around the pericardiophrenic angle in extended thymectomy and thymothymectomy for patients with myasthenia gravis (MG) and thymoma complicated by MG, respectively. Therefore, we decided that a modification was required for more accurate extended thymectomy.

Here we describe our “lateral view preceding method.” We used lateral thoracoscopy to dissect the lower poles of the thymus and mediastinal fat around the pericardiophrenic angle along the phrenic nerves preceding robot-assisted thymothymectomy.

## Technique

**Figure 1:**
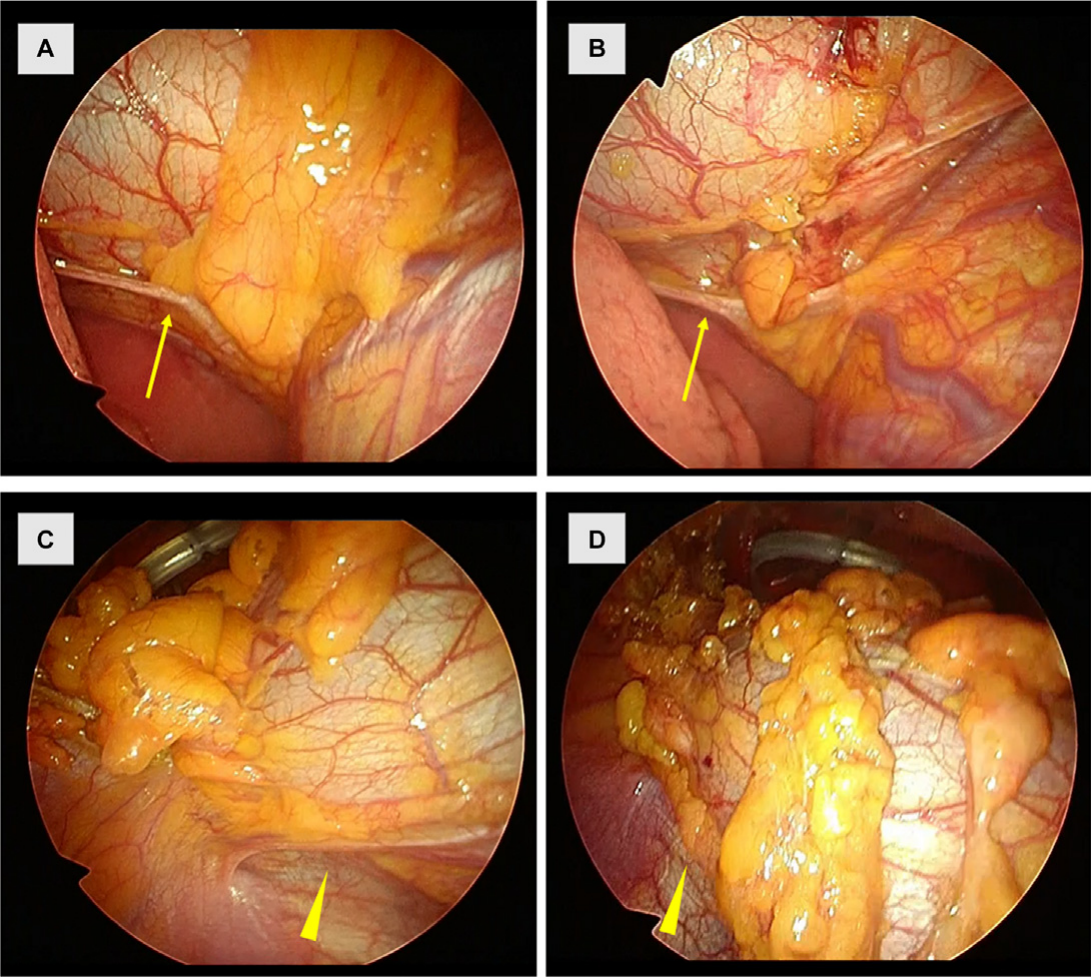
(A) Inferior section of the right phrenic nerve from the right lateral view. (B) The right lower pole of the thymus and mediastinal fat are dissected. The arrows in A and B indicate the right phrenic nerve. (C) Inferior section of the left phrenic nerve from the left lateral view. (D) The left lower pole of the thymus and mediastinal fat are dissected. The arrowheads in C and D indicate the left phrenic nerve.

A 43-year-old man with a thymoma and MG underwent minimally invasive extended thymothymectomy by the lateral view preceding method (Video). He was placed in the supine position with open legs. We made a 3-cm median skin incision at the subxiphoid, cut along the white line of the rectus abdominis muscle attached to the xiphoid, and performed blind blunt dissection of the retrosternal space with a finger. We placed a multihole wound retractor (Alnote Lapsingle, Alfresa Pharma) into the incision and started CO_2_ insufflation at 8 mm Hg by using the AirSeal Intelligent Flow System (CONMED). Under 30° endoscopy, a curved, long hook electrode opened the right and left thoracic cavities through the retrosternal space. Four 8-mm ports were placed as follows: in the left sixth intercostal space on the midclavicular and midaxillary lines, in the right sixth intercostal space on the anterior axillary line, and in 1 hole of the multihole wound retractor of the subxiphoid. The endoscope was inserted through the port at the right sixth intercostal space to obtain a clear lateral view of the right thoracic cavity. This helped us dissect the right lower pole of the thymus and mediastinal fat around the pericardiophrenic angle along the right phrenic nerve from the pericardium (Figures 1A, 1B). Forceps for grasping and a hook electrode or advanced bipolar device for dissecting were used mainly through the subxiphoid multihole wound retractor. Similarly, under a lateral endoscopic view of the left thoracic cavity through the port on the left midaxillary line, we dissected the left lower pole of the thymus and mediastinal fat around the pericardiophrenic angle along the left phrenic nerve (Figures 1C, 1D). This completed the preceding process comprising removal of the lower poles of the thymus and mediastinal fat through the lateral thoracoscopic view.

**Figure 2:**
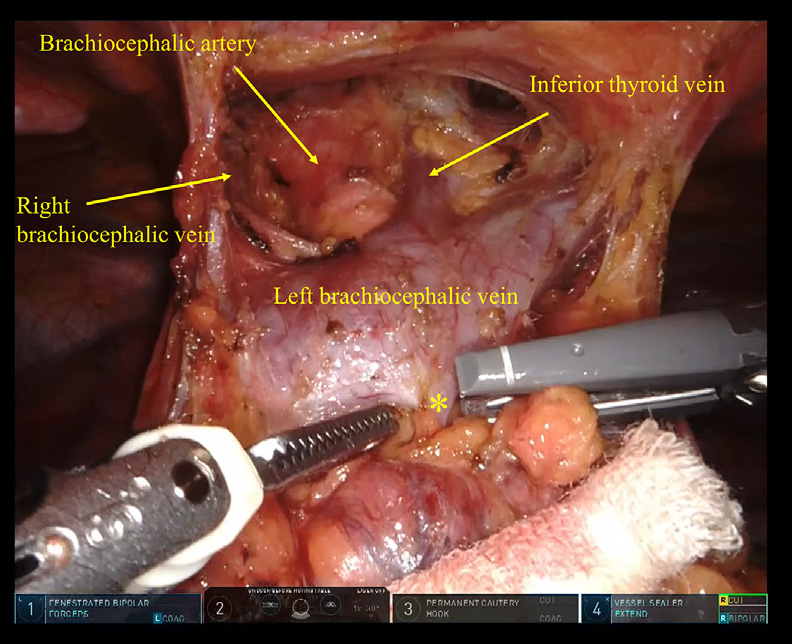
Left and right superior poles of the thymus and mediastinal fat are dissected to expose the left and right brachiocephalic veins, brachiocephalic artery, inferior thyroid vein, and trachea. The thymic vein (asterisk) is cut with a vessel-sealing device.

Next, we started the robot-assisted thymothymectomy with the Da Vinci X Surgical System (Intuitive Surgical Inc). The pleura was cut along the internal thoracic veins up to their bifurcation, and the retrosternal space was dissected to expose the left BCV and the inferior thyroid vein. Next, the upper poles of the thymus and mediastinal fat were dissected from their surrounding structures (right BCV, brachiocephalic artery, and trachea). The thymic vein was exposed and cut with a Vessel Sealer Extend device (Intuitive Surgical Inc) (Figure 2). The thymus was then dissected from the pericardium, and the mediastinal fat was cut along the anterior border of the phrenic nerves to complete the extended thymothymectomy. The specimen was placed in a retrieval bag (Inzii Retrieval Systems, Applied Medical Inc) and was removed through the subxiphoid port. The operative time was 231 minutes (39 minutes for the preceding process through the lateral thoracoscopic view and 145 minutes for the robotic console time, with the remaining 47 minutes for port placement and wound suturing), and blood loss was 5 mL. The patient was discharged on postoperative day 5 without complications or worsening of MG symptoms.

## Comment

Our proposed lateral view preceding method provided excellent visibility of the operative area and high surgical accuracy for the extended thymothymectomy. Given that minimally invasive thoracic surgery is increasing worldwide, various approaches have been proposed for resection of anterior mediastinal tumors, including thymoma.^1^, ^2^, ^3^, ^4^, ^5^ Among them, the robot-assisted 1 subxiphoid and 3 intercostal ports approach has advantages such as excellent visibility with high magnification around the upper poles of the thymus, left BCV, and superior sections of the phrenic nerves. The view of the operative area is similar to that using a median sternotomy with a bird’s eye view of the mediastinum from above.^4^ The forceps of the robotic arms have a high degree of freedom of articulation even in a narrow space beyond the left BCV. This allows for safe and highly accurate resection of mediastinal fat around the upper poles of the thymus and cutting of the thymic vein in a desirable direction without undue tension. However, this approach has disadvantages regarding the visibility of the inferior sections of the phrenic nerves and maneuverability around the pericardiophrenic angle. In contrast, a sequential bilateral thoracoscopic approach provides good visibility of both the superior and inferior sections of the phrenic nerves. Nonetheless, the view and maneuverability beyond the left BCV, especially in the area close to the inferior poles of the thyroid gland, are poor.^5^ Therefore, we proposed this lateral view preceding method, in which the advantages of each technique compensated for the characteristic disadvantages of the other, and their combination helped achieve successful extended thymothymectomy.

We recommend the following practice tips for this technique: Under the lateral thoracoscopic process, the forceps and energy devices should be used mainly through the subxiphoid multiport retractor, with countertraction through the contralateral port. In the robot-assisted process, dissection of the retrosternal space should be broadly cephalad because this can lead to improved visualization of anatomic structures. Next, dissection of both upper poles of the thymus and peripheral mediastinal fat can provide sufficient exposure of the left BCV that could help identify and cut the thymic vein. Finally, the run of the phrenic nerves should be made visible by grasping and pulling the mediastinal pleura on the resected side. This maneuver helps cut the mediastinal fat along the nerves accurately.
